# Unraveling the Mitochondrial Blueprint: Genome Characterization and Phylogenetic Insights of the Endemic Fish *Onychostoma virgulatum* (Teleostei: Cyprinidae)

**DOI:** 10.3390/genes16050541

**Published:** 2025-04-30

**Authors:** Yuting Hu, Guoqing Duan, Huaxing Zhou, Huan Wang, Amei Liu

**Affiliations:** 1 Fishery Institute of Anhui Academy of Agricultural Sciences, Hefei 230031, China; duangq2010@126.com (G.D.); hxzhou1987@126.com (H.Z.); wanghuan0325@126.com (H.W.); liuamei1205@126.com (A.L.); 2 Anhui Province Key Laboratory of Aquaculture & Stock Enhancement, Hefei 230031, China

**Keywords:** *Onychostoma virgulatum*, mitogenome, Acrossocheilinae, phylogenetic relationship, nonmonophyletic

## Abstract

**Background:** *Onychostoma virgulatum* is an endemic freshwater fish in South China, first described as a new species in 2009. However, little is known about this species and no complete mitochondrial genomes of *O. virgulatum* has been reported to date. This study seeks to elucidate the characteristics of the mitochondrial genome of *O. virgulatum* and investigate the phylogenetic relationships within the Acrossocheilinae subfamily, particularly among the genera *Onychostoma*, *Acrossocheilus*, and *Folifer*. **Methods**: The mitochondrial genome of *O. virgulatum* was sequenced and assembled. We analyzed its sequence length, nucleotide composition, and evolutionary relationships within the Acrossocheilinae by incorporate data from 58 previously published mitochondrial genomes. **Results**: The complete circular sequence is 16,606 bp in length and contains 13 protein-coding genes, 2 rRNA genes, 22 tRNA genes, and a typical control region (D-loop), all arranged in a typical order. The genomic base composition is biased toward A+T content (56.5%), with 31.4% A, 25.1% T, 27.4% C, and 16.1% G. Among about 30 Acrossocheilina species, the nonsynonymous (Ka) to synonymous substitutions (Ks) for all 13 protein-coding genes (PCGs) are significantly less than 1, suggesting strong negative or purifying selection in these species. The phylogenetic trees inferred from the mitogenome and 13 PCGs of 58 Acrossocheilinae sequences consistently indicate that: (1) *O. virgulatum* shares the closest genetic relationship with *Onychostoma barbatulum*; (2) Acrossocheilinae species are clustered into three major clades, with neither *Acrossocheilus* nor *Onychostoma* forming monophyletic groups. **Conclusions**: This study provides new insights into the taxonomy and phylogenetic relationships of Acrossocheilinae, particularly *O. virgulatum*, contributing to a better understanding of the systematics, origin, and evolution of this subfamily.

## 1. Introduction

The endemic Chinese and Southeast Asian genus *Onychostoma* Günther, 1896, is a speciose group of the subfamily Acrossocheilinae (Teleostei: Cyprinidae) [1,2,3], which was long classified under the subfamily Barbinae. It comprises small and medium-sized benthic freshwater fish with omnivorous habits and is often found in cooler, fast-flowing mountain streams and rivers [4,5,6,7,8,9,10,11,12,13] (Fishbase: https://www.fishbase.org, accessed on 10 April 2025). The species distribution within the genus *Onychostoma* is relatively complex, ranging from widely distributed species and those with restricted ranges. Most species possess significant economic value. Currently, the genus comprises 23 valid species, recorded across China, Vietnam, Thailand, Laos, and Cambodia. China is the center of diversity, hosting 19 species—12 of which are endemic [4,5,6,7,8,9,10,11,12,13] (Fishbase: https://www.fishbase.org, accessed on 10 April 2025). The taxonomic validity of this genus has long been debated. Traditionally, species identification within *Onychostoma* has relied primarily on morphological traits, including the length, size, and structure of the last unbranched (simple) dorsal fin ray as well as the shape and width of the mouth opening. However, these morphological features exhibit adaptive evolution to flowing-water habitats. Additionally, some species display sexual dimorphism in mouth morphology and ontogenetic variations in body coloration. Undoubtedly, these differences complicate species delineation and the taxonomy of certain fish within this genus remains contentious. Previous studies have confirmed the nonmonophyletic nature of *Onychostoma* and its closer relationship with the other two genera (*Acrossocheilus* and *Folifer*) in Acrossocheilinae [14,15,16,17,18,19,20]. However, a definitive classification at the generic level has yet to be established.

**Figure 1:**
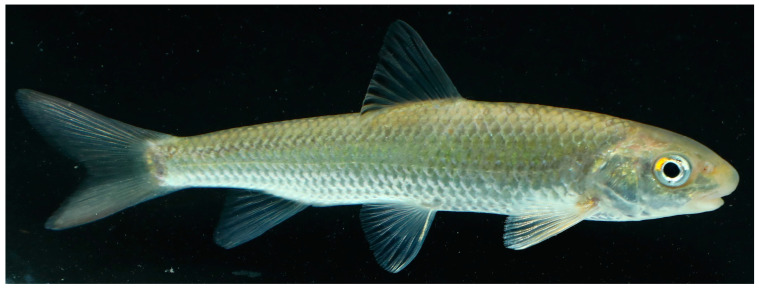
The appearance of *Onychostoma virgulatum*.

Xin, Zhang, and Cao, 2009, described *Onychostoma virgulatum* as a new species in 2009 [10] (Figure 1). This endemic freshwater fish has a narrow distribution range, occurring only in the upper reaches of the Qiupu River (Anhui Province) and the Suichuan River (Jiangxi Province)—tributaries of the southern bank of the lower Yangtze River in South China [10,21]. However, according to *The Fishes of Jiangxi* (2024) [22], specimens originally identified as *Varicorhinus* (*Onychostoma*) *lini* (Wu) from the Ganjiang, Xinjiang, and Xunwu Rivers [23,24] should be reclassified as *O. virgulatum*. Notably, the Xunwu River belongs to the upper reaches of the Dongjiang River (Pear River system), whereas the Ganjiang and Xinjiang Rivers are tributaries of the lower Yangtze River.

The species appears to exhibit a patchy distribution limited to a few areas, a pattern consistent with many Acrossocheilinae fishes [4,5,6,7,8,9,10,11,12,13,16]. However, current knowledge about the species remains limited and no complete mitochondrial genomes of *O. virgulatum* have been reported to date. This study aims to supplement the mitochondrial DNA (mtDNA) sequence data of *O. virgulatum* and, by integrating the existing relevant data, investigate the phylogenetic relationships within the Acrossocheilinae subfamily—*Onychostoma*, *Acrossocheilus*, and *Folifer*.

## 2. Materials and Methods

### 2.1. Sampling, DNA Extraction, PCR Amplification, and Sequencing

In August 2024, ten individuals of *O. virgulatum* were collected using a net from the Qiupu River (30°11′25″ N, 117°40′47″ E) in Shitai County, Anhui Province, China—the type locality of the species. All individuals were brought back to the laboratory in oxygen bags for aquaculture and molecular biology experiments. From these, one individual was randomly selected as the sample, deeply anesthetized with 100 mg/L MS-222 (Sigma, St. Louis, MO, USA), then fixed and stored in 100% ethanol and stored in the Fishery Institute of Anhui Academy of Agricultural Sciences. The genomic DNA was extracted from the muscle tissue of the sample using a DNeasy Blood and Tissue Kit (Qiagen, Dusseldorf, Germany) and then kept at −20 °C for PCR amplification.

According to references [25,26,27], a total of 18 primer pairs (Table S1) were selected to amplify the mitogenome sequences. The amplifications were performed in 25 μL reaction mixtures containing 2.5 μL 10 × buffer (Mg^2+^), 2 μL dNTPs(10 mmol/L), 1 µL of each primer (10 µmol/L), 1 U Taq polymerase (TaKaRa, Dalian, China), and approximately 100 ng template genomic DNA. PCR conditions were as follows: initial denaturation at 95 °C for 5 min, then 35 cycles of denaturation (95 °C for 35 s, 51–55 °C for 45 s, and 72 °C for 1 min), and final extension at 72 °C for 10 min. Subsequently, the targeted fragments were purified and directly sequenced in both directions by the Sangon Biotechnology Company (Sangon, Shanghai, China) after 1% agarose gel electrophoresis.

### 2.2. Genome Assembly, Annotation, and Selection Pressure Analysis

The DNA sequence fragments were manually assembled by the software Chromas 2.6.6 (http://technelysium.com.au/, accessed on 12 December 2024), Clustal X (http://www.clustal.org/, accessed on 12 December 2024), and Seaview 4.4.2 [28]. The protein-coding gene, tRNA gene, rRNA genes, and D-loop region were identified by comparing the mitogenome sequences with other *Onychostoma* sequences (downloaded from Genbank) and through homology searches. A circular map of the mitochondrial genome was generated using the Proksee server (https://proksee.ca/, accessed on 21 February 2025). The AT-skew and GC-skew were calculated using the following general formulae: AT-skew = (A% − T%)/(A% + T%) and GC-skew = (G% − C%)/(G% + C%), respectively [29]. The base composition, nucleotide substitution, and relative synonymous codon usage (RSCU) of each protein-coding gene (PCG) were determined using MEGA 12.0.9 [30], and the column diagram of RSCU was generated by the software suite PhyloSuite 1.2.3 [31].

The mean nonsynonymous (Ka), synonymous substitutions (Ks), and ratio of Ka/Ks in each PCG of 29 reference sequences within the Acrossocheilinae and mitogenome of *O. virgulatum* were calculated by DnaSP 6.0 [32], then the obtained data were used to make a column chart in Microsoft Excel.

### 2.3. Phylogenetic Analysis

We retrieved and downloaded all currently available complete mitochondrial genomes of all Acrossocheilinae species and *Spinibarbus sinensis* from the GenBank database. By PhyloSuite 1.2.3 [31], 59 sequences were reserved after deleting redundant sequences and were aligned using MAFFT 7.505 [33] with default settings. We eventually successfully extracted the nucleotide sequences and 13 PCGs of 58 mitochondrial genomes covering about 29 species and 3 unidentified sequences, including 13 *Onychostomas* species, 14 *Acrossocheilus* species, *Folifer brevifilis*, and *S. sinensis* (Table 1). A phylogenetic tree of these Acrossocheilinae species was constructed based on the mitogenome sequences and concatenated sequences of 13 PCGs using the maximum likelihood (ML) and Bayesian (BI) methods with *S. sinensis* as the outgroup.

The best substitution model GTR+F+R3 (mitogenome) or partition model with a gene + codon partitioning strategy (allowing merging of similar partitions) (PCGs) were chosen based on the Bayesian information criterion (BIC) using the smart model selection algorithm [34,35], and the ML trees were constructed using IQ-TREE 2.4.0 software with a standard bootstrap test inferred from 1000 replicates [36,37]. The GTR model (mitogenome) or partition model (PCGs) were chosen based on the Bayesian information criterion (BIC) using the smart model selection algorithm [34,35], and the BI trees were conducted using MrBayes 3.2.8 software and two independent Markov chain Monte Carlo (MCMC) chains were run for 10 million generations and beginning with a random tree in each BI analysis [35,38]. Also, all values were sampled every 1000 generations; the first 25% of samples were used as burn-in, and the remaining trees were used to construct the data. The phylogenetic trees were edited by the online tool Interactive Tree of Life (iTOL) (https://itol.embl.de/, accessed on 26 March 2025).

## 3. Results

### 3.1. Mitochondrial Genomic Structure and Composition

**Figure 2:**
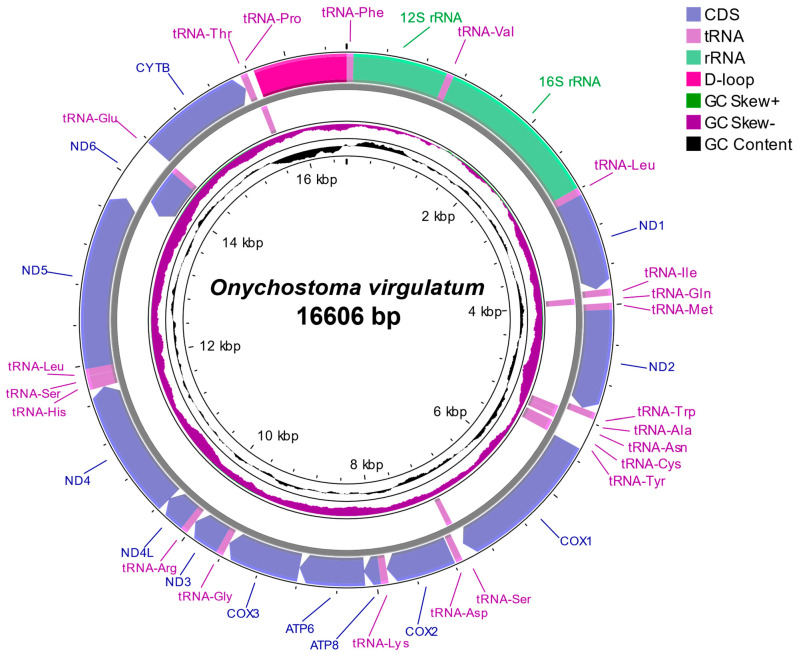
Complete mitogenome organization and gene arrangement of *O. virgulatum*.

The complete mitochondrial genome of *O. virgulatum* is a typical closed circular double-stranded DNA molecule with 16,606 bp in size and the sequence was deposited in GenBase (Accession Number: CAA104068) (Figure 2). The mitogenome typically contains 37 genes with 13 protein-coding genes, 2 ribosomal RNA (rRNA) genes, 22 tRNAs, and a control region (D-Loop) (Figure 2, Table 2). Most of the 37 genes are encoded on the H-strand but the *ND6* and 8 tRNA genes (*tRNA-Gln*, *tRNA-Ala*, *tRNA-Asn*, *tRNA-Cys*, *tRNA-Tyr*, *tRNA-Ser^(UCN)^*, *tRNA-Glu*, and *tRNA-Pro*) are encoded on the L-strand. In total, 12 of the 13 protein-coding genes of the mitogenome start with a typical ATG codon, except for the *COX1* gene (GTG codon). A total of 6 protein-coding genes end with the termination codon TAA (*ND1*, *COX1*, *ATP6*, *ND4L*, *ND5*, and *ND6*), while 6 genes terminate with a single base T, and the *COX3* gene terminates with TA. The 22 tRNA genes range in size from 67 to 76 bp, and the *tRNA-Cys* gene (67 bp) is the shortest in size, whereas the longest are the *tRNA-Leu^(UUR)^* and *tRNA-Lys* genes (76 bp). Among the 2 rRNA genes, *12S rRNA* is located between *tRNA-Phe* and *tRNA-Val* with a length of 956 bp, and *16S rRNA* is located between *tRNA-Val* and *tRNA-Leu*^(UUR)^ with a length of 1684 bp. The noncoding control region (D-loop) is located between tRNA-Pro and *tRNA-Phe* with a length of 945 bp, in which an extended terminal associated sequence (ETAS), central conserved domain (CD) including three conserved sequence blocks (CSB-F, CSB-E, and CSB-D), and conserved sequence block (CSB) consisting of three conserved sequence blocks (CSB-1, CSB-2, and CSB-3) are identified (Supplementary Figure S1). In addition, there are 11 small gene spacers (1–33 bp in size) in the mitogenome, the longest gap of which is 33 bp, located between *tRNA-Asn* and *tRNA-Cys* and 7 overlapping regions (1–7 bp in size) with the longest overlapping regions (7 bp) existing between *ATP8*/*ATP6* and *ND4L*/*ND4*. A sequence capable of initiating L-chain replication (OL) is identified in the longest gap region, which consists of 10 bases forming a conserved stem-ring structure (Figure S2).

The overall base composition of the mitogenome is 31.4% A, 25.1% T, 27.4% C, and 16.1% G, exhibiting a slight bias towards A+T content (56.5%) (Table 3). However, the D-loop exhibits a significant bias toward A+T content (66.1%), which is subject to less evolutionary selection pressure and has a fast evolutionary rate. In addition, the A+T content of the first (48.0%), second (59.0%), and third (62.1%) codon position of the PCGs are significantly different, and all PCGs except *ND6* exhibit strong anti-G bias (11.5–17.5%), especially in the second (13.7%) and third (7.3%) positions of codon.

The AT-skew of the mitogenome, rRNAs, tRNAs, D-loop, concatenated PCGs, and three PCGs (*COXI*, *ND4L*, and *ND6*) are all positive, while *ND3* is zero and nine PCGs are negative. The AT-skew of PCGs-2nd are positive but the AT-skew of PCGs-1st and PCGs-3rd is negative. Conversely, the GC-skew of mitogenome, rRNAs, D-loop, concatenated PCGs, three codon positions of PCGs, and 12 PCGs are all negative, while the tRNAs and *ND6* are positive.

### 3.2. Characteristics of Codon Usage and Selection Pressure

**Figure 3:**
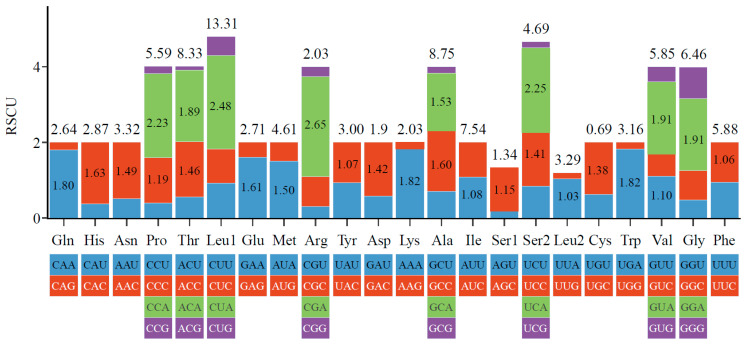
Amino acid content (top number, %) and RSCU (internal numbers) of *O. virgulatum* mitochondrial PCGs. Only RSCU values greater than 1 are displayed.

The 13 PCGs encode a total of 3794 amino acids (except for stop codons), with the highest content being 630 Leucine (16.6%) and the lowest content being 26 Cysteine (0.69%) (Figure 3). The relative synonymous codon usage (RSCU) statistics show that there are 27 preferred codons (RSCU > 1) in total, with the highest being 2.65 (Figure 3). There are 4 codons with RSCU values greater than 2: CUA, UCA, CCA, and CGA, all of which end in A.

**Figure 4:**
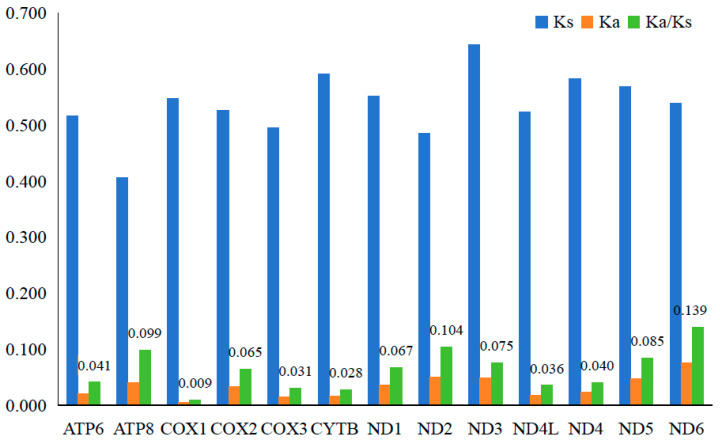
The mean Ka, Ks, and Ka/Ks values of PCGs among 30 Acrossocheilinae species.

Obviously, the Ka values (0.005–0.075) are much smaller than the Ks values (0.405–0.643) in all PCGs, and so the Ka/Ks ratio of each PCG is far less than 1, with the highest Ka/Ks ratio (0.139) in *ND6*, whereas the lowest ratio (0.009) is in *COX1* (Figure 4).

### 3.3. Phylogenetic Relationship

**Figure 5:**
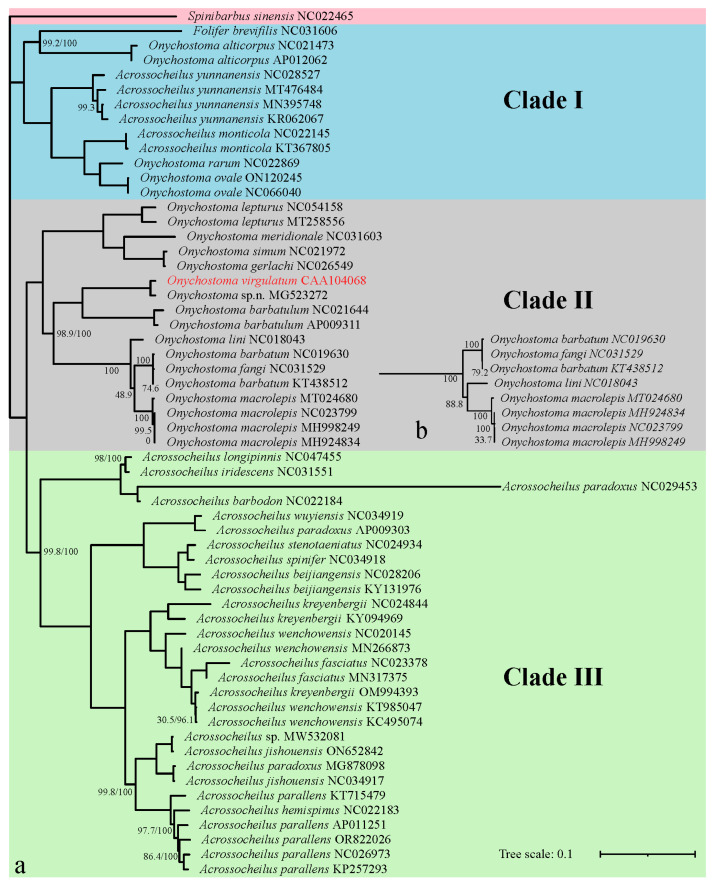
Phylogenetic trees of Acrossocheilinae based on 13 PCGs using maximum-likelihood (ML) (**a**) and Bayesian (BI) (**b**) analysis. GenBank accession number follows the species name. The numbers on nodes indicate NJ (left) and BI (right) support values, with no number indicating 100% support.

**Figure 6:**
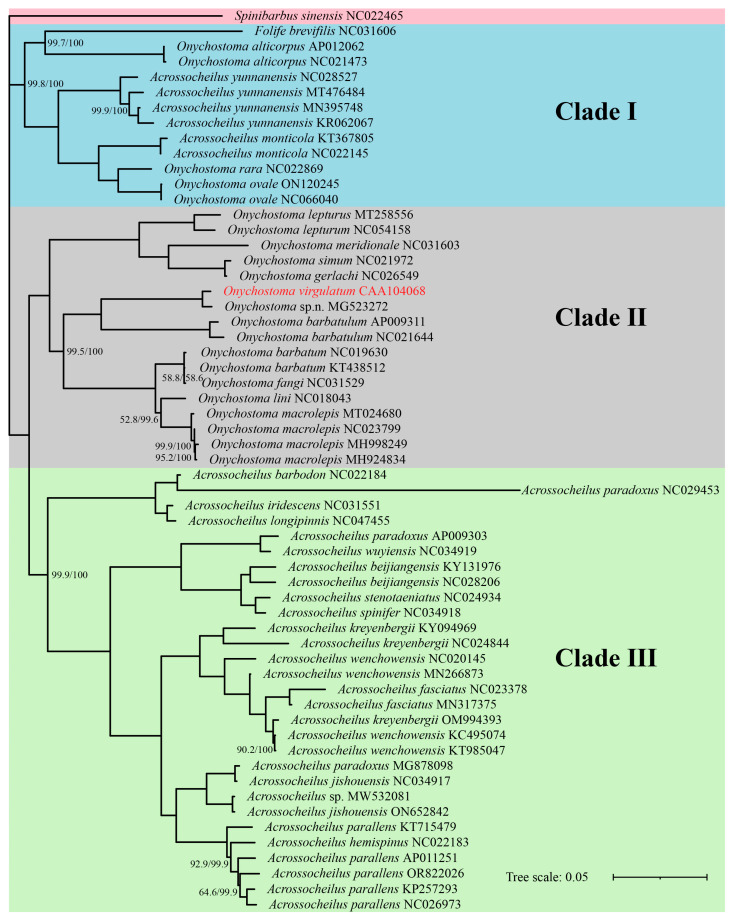
Phylogenetic trees of Acrossocheilinae based on mitogenome using maximum-likelihood (ML) and Bayesian (BI) analysis. GenBank accession number follows the species name. The two numbers on some nodes indicate ML (left) and BI (right) support values, with no number indicating 100% support.

The four phylogenetic trees obtained by ML and BI analyses are analogous with the same topologies, in which all Acrossocheilinae species are clustered into three major clades with a high node support rate (>98%) in each clade (Figure 5 and Figure 6). Clade I is considered as an ancestral group, which is a mixed clade consisting of 6 species of Acrossocheilina: (*F. brevifilis* + *Onychostoma alticorpus*) sister to (*Acrossocheilus yunnanensis* + *Acrossocheilus monticola* + sister pair (*Onychostoma ovale* + *Onychostoma rarum*)); Clade II represents the *Onychostoma* groups, including all other *Onychostoma* sequences. Clade III represents *Acrossocheilus* groups, including all other *Acrossocheilus* sequences. Clade I is sister to sister pair (Clade II + Clade III).

Additionally, these trees also consistently show that (Figure 5 and Figure 6): (1) the three sequence-group *A. yunnanensis* sequences include the unverified *A. yunnanensis* sequence (KR062067), *Onychostoma* sp. n. (MG523272) and *O. virgulatum* in the study, *Acrossocheilus* sp. (MW532081) and *Acrossocheilus jishouensis* (ON652842), are clustered into a single branch, respectively; (2) *Onychostoma simum* (NC021972) and *Onychostoma gerlachi* (NC026549) are clustered into a single branch with an abnormally close relationship; (3) the sequence identified as *Onychostoma fangi* (NC031529) is unreasonably clustered together with two *Onychostoma barbatum* sequences; (4) three sequences identified as *Acrossocheilus paradoxus* cannot be clustered in one cluster, but they are clustered with three different species, respectively; (5) *Acrossocheilus longipinnis*, a senior synonym of *Acrossocheilus stenotaeniatus* (Fishbase: https://www.fishbase.org, accessed on 10 April 2025), is clustered together with *Acrossocheilus iridescens,* while *A. stenotaeniatus* is clustered together with *Acrossocheilus spinifer*; (6) those individuals belonging to each of *Acrossocheilus fasciatus*, *Acrossocheilus kreyenbergii,* and *Acrossocheilus wenchowensis* failed to cluster according to their taxonomic circumscription; (7) the only *Acrossocheilus hemispinus* sequence (NC022183) was clustered together with the sequences of *Acrossocheilus parallens*. All the above results have a high node support value (>85%). Slight differences in the topologies among the four phylogenetic trees are observed in the positions of *O. lini* in the ML-tree based on 13 PCGs.

## 4. Discussion

We first identified the mitogenome of *O. virgulatum*, which is a typical closed circular DNA molecule with a length of 16,606 bp and which comprises 13 PCGs genes, 22 tRNA genes, 2 rRNA genes, and a typical control region (D-loop). The gene arrangements and composition exhibit similarities to those of other *Onychostoma* as well as various previously analyzed Acrossocheilinae mitogenomes [39,40,41,42]. *O. virgulatum* has long been favored by local residents, but because of habitat destruction and overfishing, the population size of the species has drastically declined in the past 10 years. It has been 15 years since the species was identified as a new species from Qiupu River in Anhui province [10], whereas the species was rarely studied because of its narrow distribution range, small population size, and semimigratory habits (wintering at the bottom of the river or in caves) [21,22]. Therefore, the determination of the mitochondrial genomes has great value, which is helpful in strengthening the conservation and management of the species.

The Ka/Ks ratio of each PCG of the Acrossocheilina mitogenome were shown to be significantly less than 1, which generally indicates strong negative or purifying selection in these species and further revealed the strong conservation of all PCGs in these species. The primary function of mitochondrial PCGs is energy production, which involves oxygen and food, so the purifying selection might be related to the common characteristics of Acrossocheilina: high dissolved oxygen demand in water and omnivorous (Fishbase: https://www.fishbase.org, accessed on 10 April 2025). The different Ka/Ks ratios among 13 genes (0.009–0.139) suggest different degrees of conservation among these genes, reflecting different degrees of functional constraints in energy metabolism. For instance, the extremely low Ka/Ks ratio in *COX1* (0.009) is significantly lower than that of *ND6* (0.139), indicating that *COX1* has undergone stronger purifying selection during evolution, meaning its amino acid sequence is highly conserved and functional mutations are strictly limited. In contrast, the relatively high Ka/Ks ratio of *ND6* suggests that it has weaker evolutionary constraints and may allow for the accumulation of more neutral or slightly deleterious mutations. Because the mitochondrial genes (such as *COX1* and *ND6*) of vertebrate are highly conserved in function, we believe that this difference mainly stems from the following evolutionary pressures [43,44,45]: *COX1*/*CYTB* are highly conserved: as they are involved in the core structure of the electron transport chain (complexes IV/III), their functions need to be strictly maintained; *ND6*/*ATP8* are prone to variation: *ND6* is the only hydrophobic protein encoded by a light chain in complex I and is susceptible to the accumulation of mutations; *ATP8* has fewer functional constraints. This difference has been found in other groups such as mammals.

Molecular phylogenies are not only used to support the placement of the newly collected specimens within the genus *Onychostoma,* even Acrossocheilina, but also to resolve the phylogenetic relationships within the group. In this study, *Onychostoma* sp.n. (MG523272) was identified as *O. virgulatum*, and they have the closest relationship with *Onychostoma barbatulum,* with a 100% support value. The sample collection location of the sequence (MG52327) was not recorded, so it is not yet possible to confirm whether there is a new distribution. The sequence of *O. barbatulum* (NC021644) was sampled from the type locality—Taiwan island. Furthermore, *O. barbatulum* is one of only two species of *Onychostoma* in Taiwan; the other is *O. alticorpus*, found only in Taiwan [46]. So, we considered the identification of both species to be credible. In addition, the unverified *A. yunnanensis* sequence (KR062067) should be *A. yunnanensis,* and *Acrossocheilus* sp. (MW532081) has an intraspecific relationship with *A. jishouensis* (ON652842) [42].

All phylogenetic trees obtained by ML and BI analyses are analogous with the same topologies, which consistently revealed the nonmonophyletic relationship between *Onychostoma* and *Acrossocheilus*, while a few species of both genera are closely related to *F. brevifilis*. This conclusion has been confirmed by many previous studies [14,15,16,17,18,19,20,25,40,41,42], such as Wang et al. (2007) [14] and Li et al. (2008) [15], who, based on RAG2 and 16S rRNA sequences, respectively, found that *Onychostoma* and *Acrossocheilus* were a nonmonophyletic group; Xin (2008) [16] carried out the taxonomic reassignment of species within *Onychostoma* based on morphological and skeletal characteristics, with a sample size more adequate than previous studies, and also found the nonmonophyly of the two genera. In recent years, several phylogenetic analyses of Acrossocheilina based on mitogenome have also come to similar conclusion [20,40,41,42]. The discovery of the nonmonophyly of the genera *Onychostoma* and *Acrossocheilus* has far-reaching implications for fish taxonomy, phylogenetic research, and biodiversity conservation, specifically manifested as: (1) The reassessment of taxonomy at the genus level in Acrossocheilus. The nonmonophyletic relationship indicates that the current definitions of genera may be based on convergent morphological features rather than true evolutionary relationships; the boundaries of genera need to be redefined in combination with molecular systematics (such as multigene or genomic data), and some species (such as *O. alticorpus*) may need to be established in new genera. (2) Adaptive radiation and convergent evolution: Nonmonophyly may reflect convergent evolution under similar ecological niches (such as similar jaw morphology resulting from benthic feeding). Studying the association between functional morphology and ecological adaptability can reveal the driving mechanisms of the diversification of *Onychostoma* and *Acrossocheilus* and even East Asian stream fishes. (3) Reassessment of species’ endangered status and precision in habitat management: Taxonomic changes may alter the uniqueness of certain species (for instance, *A. yunnanensis* is actually an independently evolved branch), necessitating a re-evaluation of their conservation priorities. After clarifying the true distribution range of monophyletic groups, a network of protected areas can be designed for key evolutionary units, preventing conservation gaps due to taxonomic errors.

In addition, species identification errors were found in many mitogenome sequences based on phylogenetic trees in the study. For instance, at least one sequence has been incorrectly identified between *O. fangi* (NC031529) and *O. barbatum* (NC019630, KT438512). Among the three sequences annotated as *A. paradoxus*, at least two demonstrate incongruent phylogenetic positioning, suggesting potential misidentification at the species level. There is confusion in species identification of three closely-related species: *A. fasciatus*, *A. kreyenbergii*. and *A. wenchowensis*, which have high morphological similarity and adjacent distribution. In common sense, *A. hemispinus* (NC022183) also should be attributed to the wrong species. After consulting the author who found *A. spinifer* as a new species [47] and uploaded the sequence of *A. spinifer* (NC034918), species identification of this sequence (NC034918) should be correct and *A. stenotaeniatus* (NC024934) is likely to be problematic. Given the limited species information, however, we cannot yet be sure whether *A. longipinnis* (NC047455) and *A. iridescens* (NC031551) are correct. As mentioned in the introduction, adaptive evolution to running water (convergence), sexual dimorphism, and ontogenetic variations in some morphological characters bring confusion in the delineation of these species. These results highlight the limitations of classification based solely on morphology. It should be noted that there are species identification errors in many taxon in the mitochondrial public data. Therefore, to correctly identify species and further ensure the accuracy of mitochondrial data in public databases (such as GenBank and GenBase), we propose a checklist for quality control of public mitochondrial data as follows: (1) Improve basic information: clearly define the submitted information of samples (required fields: species, specimen photos, geographical location, collection time, and contact information of the submitter) and record the DNA extraction and sequencing methods. (2) Taxonomic validation: combined with integrative taxonomy [48]: provide morphological evidence, additional nuclear gene markers that support the consistency of mitochondrial data and ecological niche modeling that check whether the geographical distribution of the samples matches the ecological niches of the known species. (3) Data quality assessment: check sequence integrity and consistency, establish a public platform, or utilize a database feedback system to flag suspicious data. (4) Long-term maintenance: regular review: database managers collaborate with taxonomists to re-evaluate disputed data and encourage users to supplement missing information. It is fortunate that these errors are only found in species within the genus from clade II or clade III. It suggests, at the very least, that the species identification between genera is clear, but that the definition at the genus level is problematic, as mentioned above, which needs more new genus in Acrossocheilina.

Limited information is currently available regarding the mitochondrial genome of some species of Acrossocheilina. Therefore, further investigations incorporating extensive taxon sampling are imperative to accurately validate the phylogenetic connections within the genera *Onychostoma*, *Acrossocheilus*. Our findings significantly contribute to the study of the genetic diversity and taxonomic status of *O. virgulatum*, also offering valuable insights to better understand the evolution of Acrossocheilina.

## Figures and Tables

**Table 1:** Information on 59 mitogenome sequences of Acrossocheilinae used in this study.

NO.	Valid Species	Author	Accession NO.	Size(bp)	AT(%)	AT-Skew	GC-Skew
1	*Acrossocheilus beijiangensis*	Wu and Lin, 1977	NC028206	16,600	56.2	0.11	−0.26
			KY131976	16,596	55.9	0.11	−0.27
2	*Acrossocheilus fasciatus*	Steindachner, 1892	NC023378	16,589	55.7	0.11	−0.26
			MN317375	16,589	55.7	0.11	−0.26
3	*Acrossocheilus hemispinus*	Nichols, 1925	NC022183	16,590	55.9	0.12	−0.27
4	*Acrossocheilus iridescens* (*Acrossocheilus barbodon*)	Nichols and Pope, 1927	NC031551	16,596	55.9	0.13	−0.28
			NC022184	16,596	55.9	0.13	−0.28
5	*Acrossocheilus jishouensis*	Zhao, Chen, and Li, 1997	NC034917	16,587	56.3	0.11	−0.26
			ON652842	16,594	56.3	0.11	−0.26
6	*Acrossocheilus kreyenbergii*	Regan, 1908	NC024844	16,849	56.6	0.10	−0.25
			KY094969	16,596	56.0	0.10	−0.25
			OM994393	16,592	55.8	0.11	−0.26
7	*Acrossocheilus longipinnis* (*Acrossocheilus stenotaeniatus*)	Wu, 1939	NC047455	16,593	55.9	0.13	−0.27
			NC024934	16,594	55.9	0.12	−0.27
8	*Acrossocheilus monticola*	Günther, 1888	NC022145	16,599	55.9	0.12	−0.28
			KT367805	16,605	55.9	0.12	−0.28
9	*Acrossocheilus paradoxus*	Günther, 1868	NC029453	16,597	54.8	0.07	−0.24
			AP009303	16,595	55.6	0.11	−0.26
			MG878098	16,586	56.2	0.11	−0.26
10	*Acrossocheilus parallens*	Nichols, 1931	NC026973	16,592	55.7	0.11	−0.26
			AP011251	16,590	55.6	0.11	−0.26
			KP257293	16,588	55.7	0.11	−0.26
			KT715479	16,591	55.5	0.11	−0.26
			OR822026	16,589	55.5	0.11	−0.26
11	*Acrossocheilus spinifer*	Yuan, Wu, and Zhang, 2006	NC034918	16,591	55.9	0.12	−0.27
12	*Acrossocheilus wenchowensis*	Wang, 1935	NC020145	16,591	55.8	0.11	−0.26
			KC495074	16,591	55.8	0.11	−0.26
			KT985047	16,591	55.8	0.11	−0.26
			MN266873	16,593	56.0	0.11	−0.26
13	*Acrossocheilus wuyiensis*	Wu and Chen, 1981	NC034919	16,594	55.8	0.11	−0.27
14	*Acrossocheilus yunnanensis*	Regan, 1904	NC028527	16,588	56.1	0.12	−0.27
			MN395748	16,590	56.1	0.12	−0.27
			MT476484	16,587	56.0	0.12	−0.27
			KR062067 *	16,596	56.2	0.12	−0.26
15	*Acrossocheilus* sp.		MW532081 *	16,594	56.3	0.11	−0.26
16	*Onychostoma alticorpus*	Oshima, 1920	AP012062	16,604	54.5	0.13	−0.27
			NC021473	16,607	54.5	0.13	−0.27
17	*Onychostoma barbatulum*	Pellegrin, 1908	AP009311	16,612	56.7	0.11	−0.27
			NC021644	16,597	56.6	0.11	−0.26
18	*Onychostoma barbatum*	Lin,1931	KT438512	16,589	56.0	0.13	−0.28
			NC019630	16,592	56.0	0.13	−0.28
19	*Onychostoma fangi*	Kottelat, 2000	NC031529	16,597	56.0	0.13	−0.28
20	*Onychostoma gerlachi*	Peters, 1881	NC026549	16,601	55.6	0.13	−0.27
21	*Onychostoma lepturus*	Boulenger, 1900	MT258556	16,598	55.3	0.13	−0.28
			NC054158	16,601	55.2	0.13	−0.28
22	*Onychostoma lini*	Wu, 1939	NC018043	16,595	56.2	0.12	−0.27
23	*Onychostoma macrolepis*	Bleeker, 1871	MH924834	16,594	55.7	0.12	−0.27
			MH998249	16,621	55.8	0.12	−0.27
			MT024680	16,597	55.8	0.12	−0.27
			NC023799	16,595	55.8	0.12	−0.27
24	*Onychostoma meridionale*	Kottelat, 1998	NC031603	16,595	55.5	0.12	−0.27
25	*Onychostoma ovale*	Pellegrin and Chevey, 1936	NC066040	16,602	55.6	0.13	−0.28
			ON120245	16,600	55.6	0.13	−0.28
26	*Onychostoma rarum*	Lin, 1933	NC022869	16,590	55.7	0.13	−0.28
27	*Onychostoma simum*	Sauvage and Dabry de Thiersant, 1874	NC021972	16,601	55.6	0.13	−0.27
28	*Onychostoma* sp. n.		MG523272 *	16,602	56.5	0.11	−0.26
29	*Onychostoma virgulatum*	Xin, Zhang, and Cao, 2009	CAA104068 ^a^	16,606	56.5	0.11	−0.26
30	*Folifer brevifilis*	Peters, 1881	NC031606	16,707	55.7	0.12	−0.26
31	*Spinibarbus sinensis*	Bleeker, 1871	NC022465	16,591	57.4	0.11	−0.25

* The species name of sequence are unverified; ^a^ the sequence obtained in this study; the contents in parentheses are junior synonym. All author information are cited from Fishbase (https://www.fishbase.org, accessed on 10 April 2025).

**Table 2:** Composition and structure of *O. virgulatum* mitochondrial genome.

Number	Genes	Location (bp)	Size (bp)	Intergenic Spacer (bp)	Coding Strand	Condon	
						Start	End
1	*tRNA-Phe*	1–69	69	0	H		
2	*12S rRNA*	70–1025	956	0	H		
3	*tRNA-Val*	1026–1097	72	0	H		
4	*16S rRNA*	1098–2781	1684	0	H		
5	*tRNA-Leu*	2782–2857	76	0	H		
6	*ND1*	2858–3832	975	6	H	ATG	TAA
7	*tRNA-Ile*	3839–3909	71	−2	H		
8	*tRNA-Gln*	3978–3908	71	3	L		
9	*tRNA-Met*	3982–4050	69	0	H		
10	*ND2*	4051–5095	1045	0	H	ATG	T--
11	*tRNA-Trp*	5096–5165	70	2	H		
12	*tRNA-Ala*	5237–5168	70	1	L		
13	*tRNA-Asn*	5311–5239	73	33	L		
14	*tRNA-Cys*	5411–5345	67	−1	L		
15	*tRNA-Tyr*	5481–5411	71	1	L		
16	*COX1*	5483–7033	1551	0	H	GTG	TAA
17	*tRNA-Ser*	7104–7034	71	5	L		
18	*tRNA-Asp*	7110–7179	70	13	H		
19	*COX2*	7193–7883	691	0	H	ATG	T--
20	*tRNA-Lys*	7884–7959	76	1	H		
21	*ATP8*	7961–8125	165	−7	H	ATG	TAG
22	*ATP6*	8119–8802	684	−1	H	ATG	TAA
23	*COX3*	8802–9586	785	0	H	ATG	TA-
24	*tRNA-Gly*	9587–9659	73	0	H		
25	*ND3*	9660–10,008	349	0	H	ATG	T--
26	*tRNA-Arg*	10,009–10,079	71	0	H		
27	*ND4L*	10,080–10,376	297	−7	H	ATG	TAA
28	*ND4*	10,370–11,750	1381	0	H	ATG	T--
29	*tRNA-His*	11,751–11,819	69	0	H		
30	*tRNA-Ser*	11,820–11,888	69	1	H		
31	*tRNA-Leu*	11,890–11,962	73	0	H		
32	*ND5*	11,963–13,786	1824	−4	H	ATG	TAA
33	*ND6*	14,304–13,783	522	0	L	ATG	TAA
34	*tRNA-Glu*	14,373–14,305	69	5	L		
35	*CYTB*	14,379–15,519	1141	0	H	ATG	T--
36	*tRNA-Thr*	15,520–15,591	72	−1	H		
37	*tRNA-Pro*	15,661–15,591	71	0	L		
38	D-loop	15,662–16,606	945	0	H		

Note: H is heavy chain and L is light chain.

**Table 3:** Nucleotide compositions of different regions of *O. virgulatum*.

Regions	Size (bp)	T (U)	C	A	G	AT (%)	AT-Skew	GC-Skew
Full genome	16,606	25.1	27.4	31.4	16.1	56.5	0.112	−0.262
D-loop	945	32.6	21.1	33.5	12.8	66.1	0.014	−0.245
PCGs	11,403	27.0	28.1	29.4	15.5	56.4	0.042	−0.288
tRNAs	1563	26.8	21.4	28.0	23.8	54.8	0.022	0.054
rRNAs	2640	20.1	24.5	34.7	20.7	54.8	0.266	−0.085
PCGs-1st	3801	20.9	26.4	27.1	25.6	48.0	0.130	−0.015
PCGs-2nd	3801	40.4	27.3	18.6	13.7	59.0	−0.369	−0.333
PCGs-3rd	3801	19.7	30.6	42.4	7.3	62.1	0.365	−0.613
*ATP6*	684	27.2	28.4	30.6	13.9	57.8	0.058	−0.343
*ATP8*	165	23.6	28.5	36.4	11.5	60.0	0.212	−0.424
*COX1*	1551	28.9	26.4	27.2	17.5	56.1	−0.030	−0.201
*COX2*	691	26.5	26.6	30.7	16.2	57.2	0.073	−0.243
*COX3*	785	26.5	28.9	27.5	17.1	54.0	0.019	−0.258
*CYTB*	1141	28.1	28.4	28.3	15.2	56.4	0.003	−0.304
*ND1*	975	26.1	29.6	29.1	15.2	55.2	0.056	−0.323
*ND2*	1045	23.2	30.4	34.1	12.3	57.3	0.191	−0.423
*ND3*	349	28.4	29.2	28.4	14.0	56.8	0.000	−0.351
*ND4*	1381	25.6	29.2	31.2	14.0	56.8	0.098	−0.352
*ND4L*	297	29.0	28.3	26.9	15.8	55.9	−0.036	−0.282
*ND5*	1824	24.7	30.3	32.4	12.6	57.1	0.135	−0.413
*ND6*	522	41.0	13.2	13.0	32.8	54.0	−0.518	0.425
*16S rRNA*	1684	20.6	23.1	36.3	20.0	56.9	0.276	−0.072
*12S rRNA*	956	19.2	27.0	31.9	21.9	51.1	0.247	−0.105

## Data Availability

The mitogenome sequence of *O. virgulatum* supporting the findings of this study has been deposited in the GenBase database (https://ngdc.cncb.ac.cn/genbase/) under the accession number CAA104068. The sequence will be made openly available starting 29 May 2025.

## Supplementary Materials

The following supporting information can be downloaded at: https://www.mdpi.com/article/10.3390/genes16050541/s1, Figure S1: the structure and sequence of the control region of the *O. virgulatum* mitochondrial genome; Figure S2: stem-loop structure of L-strand replication initiation region of *O. virgulatum*; Table S1: sequences of primers used in amplification of the complete mitochondrial genome in *O. virgulatum*.
