# Supplementary material for: Unraveling the Mitochondrial Blueprint: Genome Characterization and Phylogenetic Insights of the Endemic Fish Onychostoma virgulatum (Teleostei: Cyprinidae)
Source: Genes (Basel). 2025 Apr 30;16(5):541. doi: 10.3390/genes16050541 (PMC12111418; doi:10.3390/genes16050541)
Supplement: Supplementary file 1 [file genes-16-00541-s001.zip › Figure S1. The structure and sequence of the control region of O. virgulatum mitogenome.pdf]

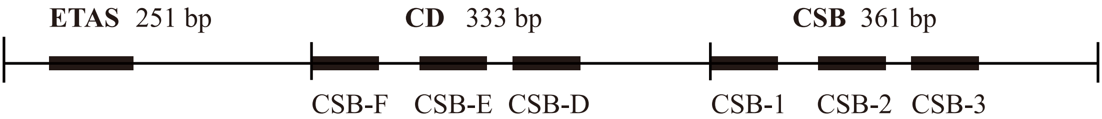

AATAAACCATTCCTTATGGTTTACTATATAATATGCCTAATTT**TACAT**TGATGTGTCAA**TACAT**CT**ATGTA**
TTATCACCAGTCCATTATTTAACCACAAAGCAAGTACTAATTACTAAGCTATACATAAAGCATAATATT

ETAS

AAGACTCACAAACTCAGTCATTTTAACCCGGGTAATTTATTAATCCCTAAAAAATTGTCCTCAAATTTTT
CCTTGAAATAATCAACTAGTATCCCACTAAATACTTCTTA**ATGTAGTAAGAAACCACC**AACCAGTTTAT

CSB-F

ATAAAGGTACATCATGCATGATAGAATC**AGGGACAATAATCGTGGGGG**TCGCACAATATGAAC**TATTA**

CSB-E

CTGGCATCTGGTTCCTATTTCAGGAACATAACTGCATTATCCCATCCTCGGATAATTATACTGGCATCT

CSB-D

GATTAAGATTAATGGTGTGGTACATATGTCTCGTTACCCACCATGCCGAGCGTTCTTTATATGCATAAC
GTTATTTTTTTTGGTTTCCTTTCATTTTGCATCTCAGAGTGCAGGCACAAATGTTAATTTAAGGTAGAA
CATTTTCCTTGTATGTCATGATAAATA**TTAATTATTGAAAGACATA**ACTTAAGAACTACATACTATTTAA

CSB-1

TCAAGTGCATAACATATTCATCTCTTGTTCAATTATCCTTGTATATATACCCCCTTCTGGTTTTTGCGCGA
CAAACCCCCCTACCCCCCTACGCCCAGCGAATCCTGTTATTCT**TGTCAAACCCCGAAACCAA**GAAG

CSB-2 **CSB-3**

GACCCAAGAACGCACGAGCCAACAAGTTGAAGTATGAATTGGCATCCCCATTATATATATATATATATATA
TATATATGCATCAATTTTTATTTTTTTCCGCTTCCACCAAAAACCTAAAAGCCCCTACCAAAAATTGATG
GAAGAATAACCCGACACTAAATATTCTAACATATTGATAA
