# Supplementary material for: Unraveling the Mitochondrial Blueprint: Genome Characterization and Phylogenetic Insights of the Endemic Fish Onychostoma virgulatum (Teleostei: Cyprinidae)
Source: Genes (Basel). 2025 Apr 30;16(5):541. doi: 10.3390/genes16050541 (PMC12111418; doi:10.3390/genes16050541)
Supplement: Supplementary file 1 [file genes-16-00541-s001.zip › Table S1. Sequences of primers used in amplification of the complete mitochondrial genome in Onychostoma virgulatum.pdf]

Supplementary Table:

Table S1. Sequences of primers used in amplification of the complete mitochondrial genome in *Onychostoma virgulatum*

Number	Primer name	Primer sequences (5′—3′)	*T*a/°C
1	L1	ACTATTACTGGCATCTGGTTCC	57
	H1	ATGTTTATCACTGCTGAATTCC	
2	L2	GCACTGAAGATGCCAAGATG	50
	H2	TTCTCGGTGTAAGTGAGATGC	
3	L3	GCTCTGAGACGCGTACACAC	53
	H3	TGGCCGTTCATACAGGTCTC	
4	L4	ACAAGCCTCGCCTGTTTAC	55
	H4	GGTCCTACTACATTTGGTCC	
5	L5	AGAGGTTCAAATCCTCTTCC	50
	H5	CTTTATTAGCTGACCACGCC	
6	L6	AGGACCACTTTGATAGAGTG	52
	H6	CTACTTAGAGCTTTGAAGGC	
7	L7	CATTATCGCCACAACAATAG	55
	H7	GGTGTTGAGGTTTCGATCTG	
8	L8	GCAATCAACTTCATCACCAC	50
	H8	TGACAGAGTGGTTATGTGGC	
9	L9	CACCGCCAAACGAGAAGTAC	50
	H9	GTGGTCTGGAGTCACCAATC	
10	L10	CAATGCTCAGAAATTTGCGG	50
	H10	TGGAATCAGATTGCTAAGCC	
11	L11	CGACTTACAGCCAACCTAAC	50
	H11	ACCGTGTGATTGGAAGTCAC	
12	L12	GCTACAGGATTCCACGGACTAC	55
	H12	GGCTGGCTAGAATTATGAGTGG	
13	L13	CTAGTTGCCACAGTTCG	50
	H13	AGGTTTGGTAGTGGTGG	
14	L14	CACGGATTAGCATCTTCAGC	55
	H14	GTCCGATGTCTCCTACTCGG	
15	L15	ATCGGCTGAGAAGGAGTAGG	55
	H15	TCTATGGCTGTTAGCAGGCC	
16	L16	AACGACTTGCCTGAGGAAG	50
	H16	GTATGGCTAGGAATAGGCC	
17	L14724	GACTTGAAAAACCACCGTTG	50
	H15915	CTCCGATCTCCGGATTACAAGAC	
18	DL1	ACCCCTGGCTCCCAAAGC	55
	DH2	ATCTTAGCATCTTCAGTG	
