# Supplementary figures and images for: Unraveling the Mitochondrial Blueprint: Genome Characterization and Phylogenetic Insights of the Endemic Fish Onychostoma virgulatum (Teleostei: Cyprinidae)
Source: Genes (Basel). 2025 Apr 30;16(5):541. doi: 10.3390/genes16050541 (PMC12111418; doi:10.3390/genes16050541)

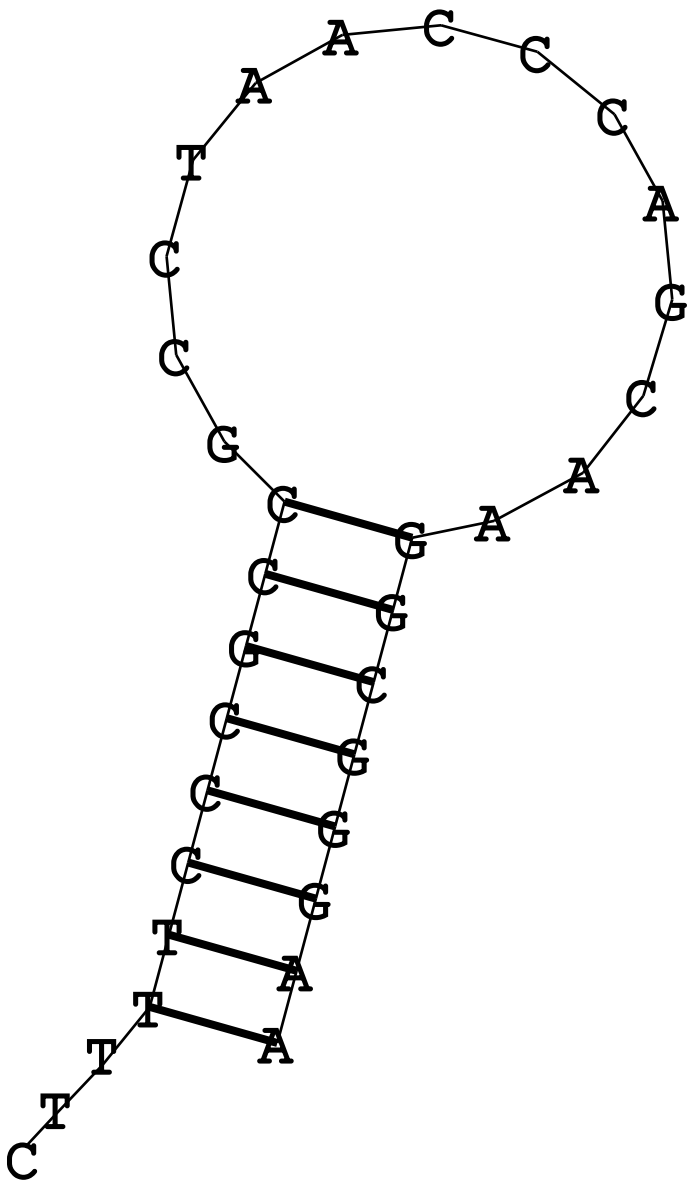

Supplement: Supplementary file 1 [file genes-16-00541-s001.zip › Figure S2. Stem-loop structure of L-strand replication initiation region.pdf]
